# NoxO1 Controls Proliferation of Colon Epithelial Cells

**DOI:** 10.3389/fimmu.2018.00973

**Published:** 2018-05-08

**Authors:** Franziska Moll, Maria Walter, Flávia Rezende, Valeska Helfinger, Estefania Vasconez, Tiago De Oliveira, Florian R. Greten, Catherine Olesch, Andreas Weigert, Heinfried H. Radeke, Katrin Schröder

**Affiliations:** ^1^Institute for Cardiovascular Physiology, Goethe-University, Frankfurt, Germany; ^2^Institute for Tumor Biology and Experimental Therapy, Georg-Speyer-Haus, Frankfurt, Germany; ^3^Institute of Biochemistry I, Faculty of Medicine, Goethe-University, Frankfurt, Germany; ^4^Pharmazentrum Frankfurt, Goethe-University, Frankfurt, Germany

**Keywords:** reactive oxygen species, colon, Nox1, NoxO1, proliferation, inflammation

## Abstract

**Aim:**

Reactive oxygen species (ROS) produced by enzymes of the NADPH oxidase family serve as second messengers for cellular signaling. Processes such as differentiation and proliferation are regulated by NADPH oxidases. In the intestine, due to the exceedingly fast and constant renewal of the epithelium both processes have to be highly controlled and balanced. Nox1 is the major NADPH oxidase expressed in the gut, and its function is regulated by cytosolic subunits such as NoxO1. We hypothesize that the NoxO1-controlled activity of Nox1 contributes to a proper epithelial homeostasis and renewal in the gut.

**Results:**

NoxO1 is highly expressed in the colon. Knockout of NoxO1 reduces the production of superoxide in colon crypts and is not subsidized by an elevated expression of its homolog p47phox. Knockout of NoxO1 increases the proliferative capacity and prevents apoptosis of colon epithelial cells. In mouse models of dextran sulfate sodium (DSS)-induced colitis and azoxymethane/DSS induced colon cancer, NoxO1 has a protective role and may influence the population of natural killer cells.

**Conclusion:**

NoxO1 affects colon epithelium homeostasis and prevents inflammation.

## Introduction

Colon is an organ with an enormous tissue turnover, i.e., a relatively high level of cell renewal. This can be even further elevated by the presence of pathogens, non-specific injury, or dietary factors such as high levels of bile acids (1). Interestingly, hyperproliferation may also serve as a host defense mechanism against invading pathogens, including gastrointestinal-dwelling nematodes. At least in the large intestine, the hyperproliferation and increased movement act as an “epithelial escalator” to expel the pathogens (2).

Reactive oxygen species (ROS) affect proliferation and differentiation of multiple cells, such as adipocytes, osteoclasts, and smooth muscle cells (3–5). A major source of controlled ROS formation is the family of NADPH oxidases. The seven members of the NADPH oxidase family, namely Nox1–5 and Duox1 and 2, differ in their cellular localization as well as in their mode of activation. While Nox1–2 need to be activated by cytosolic subunits, Nox4 appears to be constitutively active, Nox5 is activated by Ca^2+^, and the Duoxes are activated by membrane-bound subunits (6). The predominant isoforms found in the intestinal system are Nox1 and Duox2, with Nox1 being mainly expressed in the ileum, cecum, and colon, while Duox2 can be found in all compartments of the intestinal tract (7). Both Nox1 and Duox2 have been shown to play a role in the development, progression, and healing of ulcerative colitis (8–11). Nox1 contributes to ROS formation and mediates symbiosis between the gut microbiota and the intestine (12). Nox1 further regulates processes involved in homeostasis and injury repair of the colon (13, 14). Duox2 and Nox1 both build multicomponent complexes. Duox2 is associated with DuoxA2 and produces hydrogen peroxide (H_2_O_2_) in a Ca^2+^-dependent manner (15). The Nox1 complex consists of the scaffolding protein p22phox, an activating subunit NoxA1, and an organizing subunit NoxO1 (16). NoxO1 is considered to contribute to a constitutive production of ${\text{O}}_{2}^{\cdot -}$ by Nox1 and thereby contributes to a shift in cellular behavior and differentiation as shown for endothelial cells, where it mediates the maintenance of a stalk cell phenotype and limits angiogenesis (17). A role for NoxO1 in intestinal tissues has not been identified so far. In human colon cancer cells, proteasomal degradation of NoxO1 reduces the Nox1-dependent ROS formation, and expression and stability of NoxO1 were significantly increased in human colon cancer tissues compared to normal colon (18). This finding suggests a role of NoxO1 in cancer. However, whether or not NoxO1 upregulation is the cause or the consequence of colon cancer remains elusive. The same holds true for the physiological role of NoxO1 in the colon. Within the present study, we characterize the function of NoxO1 in colon homeostasis and pathology. This includes NoxO1s localization and its role in the production of ROS in the colon.

## Materials and Methods

### Animals and Animal Procedures

All animal experiments were approved by the local governmental authorities (approval number: FU1074, F28/46) and were performed in accordance with the animal protection guidelines. Knockout mice for NoxO1 (NoxO1^−/−^) were generated as previously described and bred heterozygous, to obtain wild-type (WT) and knockout littermates (17). Mice deficient of p47phox (p47phox^−/−^) were kindly provided by Ajay M Shah, Kings College London. Nox1y/^−^ mice were kindly provided by Karl-Heinz Krause (19). Mice were housed in a specified pathogen-free facility with 12/12 hours day and night cycle and free access to water and chow every time.

Colitis was induced by with 2% dextran sulfate sodium (DSS) (#16011080; MP Biomedicals) in drinking water for 5 days, with a recovery phase of 3 days and were sacrificed on day 8. Body weight and physical condition were controlled daily. For the induction of colon carcinomas, a combination of the pro-inflammatory DSS together with a single intraperitoneal injection of 10 mg/kg body weight azoxymethane (AOM, Sigma-Aldrich) was used. One week after AOM injection, three cycles of 5 days with 1.5% DSS-enriched drinking water followed by 2 weeks with usual drinking water were applied. Then, mice were sacrificed, and the colon was used for further analysis. To generate colon swiss rolls, colon was isolated, flushed, and cut longitudinally. It was then rolled from proximal to distal, fixed overnight in 4% PFA, dehydrated, and embedded in paraffin.

### Flow Cytometry

Characterization of immune cell subsets was performed essentially as described previously (20). Samples were acquired with a LSRII/Fortessa flow cytometer (BD Biosciences) and analyzed using FlowJo software Vx (Treestar). All antibodies and secondary reagents were titrated to determine optimal concentrations. CompBeads (BD) were used for single-color compensation to create multi-color compensation matrices. For gating, fluorescence minus one controls were used. The instrument calibration was controlled daily using Cytometer Setup and Tracking beads (BD). For characterization of immune cell subsets, the following antibodies were used: anti-CD3-PE-CF594, anti-CD4-BV711, anti-CD11c-AlexaFluor700, anti-CD19-APC-H7, anti-CD326-BV711, anti-Ly-6C-PerCP-Cy5.5, anti-NK1.1-BV510 (all from BD Biosciences), anti-CD8-BV650, anti-CD11b-BV605, anti-F4/80-PE-Cy7, anti-GITR-FITC, anti-Ly-6G-APC-Cy7 (from BioLegend), anti-CD31-PE-Cy7, anti-CD117-APC-eFluor780 (from eBioscience), anti-CD45-VioBlue, and anti-HLA-DR-APC (from Miltenyi).

### Histological Colitis Scoring

Sections were stained with hematoxylin and eosin according to standard protocols, and severity of colitis was assessed in a blinded way as described before (21). The colonic epithelial damage score was assigned as follows: 0, normal; 1, hyperproliferation; 2, mild-to-moderate loss of crypts, 10–50%; 3, severe loss of crypts, 50–90%; 4, complete loss of crypts, intact epithelium; and 5, ulcerated epithelium. The infiltration with inflammatory cells score was assigned separately for: mucosa (0 = normal, 1 = mild, 2 = modest, and 3 = severe), submucosa, and muscle/serosa (0 = normal, 1 = mild to modest, and 2 = severe). The scores for epithelial damage and inflammatory cell infiltration were added, resulting in a total score ranging from 0 to 12.

### RNAScope^®^ *In Situ* Hybridization

*In situ* hybridization by RNAscope^®^ technique was performed according to the manufacturer’s instructions [Advanced Cell Diagnostics (ACD), Newark, CA, USA]. Briefly, 4 μm thick sections were deparaffinized and treated with H_2_O_2_ followed by antigen retrieval and protease treatment. For murine tissue, probes for NoxO1 ACD #466541, Nox1 ACD # 464651, p47phox ACD # 481991, Nox2 ACD # 403381, Lgr5 ACD # 312171, Adgre1 ACD #460651, and positive/negative controls (peptidylprolyl isomerase B ACD #313911/*Bacillus subtilis* dihydrodipicolinate reductase ACD #310043) were used. For human samples, POLR2A-positive control ACD # 310451 and NoxO1 ACD # 482071 probes were used. Probes were hybridized for 2 h followed by six amplification steps. The signal was detected with RNAscope^®^ 2.5 HD detection kit Brown (ACD #322310) and specimens counterstained with Gill’s hematoxylin No1. For the duplex staining, probes were hybridized for 2 h followed by 10 amplification steps. The signal was detected by RNAscope^®^ 2.5 HD Duplex Reagent Kit ACD # 322430 and visualized with a light microscope.

### Immunohistochemistry

Paraffin blocks were cut, and slides were deparaffinized for further staining in descending ethanol series from 100 to 70%. For antigen retrieval, the slides were boiled 10 min in 1× antigen retrieval buffer (Dako). After blocking with 3% hydrogen peroxide for 10 min and BSA for 1 h, primary antibodies were applied over night at 4°C. For NoxO1 (non-commercial) and phospho-Histone3 (Millipore), the slides were incubated with goat-anti-rabbit HRP (Jackson immune research) secondary antibody for 2 h at room temperature. After incubation with F4/80 (AbD Serotech) primary antibody, the slides were incubated with anti-rat Histofine^®^ Simple Stain mouse MaxPO (Nichirei Biosciences) for 30 min at room temperature. Staining was developed with DAB (Vector Laboratories), and nuclei were counterstained with Gill’s No1 hematoxylin. Human samples were purchased from OriGene.

### Multiplex Immunohistochemistry and Immunofluorescence Analysis

Colon swiss rolls were stained and analyzed using the PhenOptics system with Opal^TM^ 6-Color Fluorescent IHC Kits according to the manufacturer’s instructions (Perkin-Elmer, Rodgau, Germany). The following antibodies were used for the staining: pan-cytokeratin (Pan-CK) (Abcam; ab27988), cleaved Caspase 3 (cCasp3) (Cell Signaling, #9661), Ki67 (clone: SP6) (Abcam, ab16667), and alpha-smooth muscle actin (Sigma, F3777). Nuclei were counterstained with DAPI, and the slides were mounted with Fluoromount-G (SouthernBiotech, Birmingham, AL, USA). For image acquisition at 4× and 20×, the Vectra^®^ 3 automated quantitative pathology imaging system (Perkin-Elmer) was used, and the images were analyzed using inForm2.0 Software (Perkin-Elmer).

### Crypt Isolation and Intestinal Organoids

Crypt isolation and organoid cultures from murine intestine were performed based on the methods of Mahe and colleagues (22) and using the Intesticult™ system from StemCell Technologies according to manufacturer’s instructions. Additionally, 100 ng/mL murine Wnt3a (Peprotech) was added to the medium. Colons were isolated from mice and crypts isolated as described. 700 crypts were seeded in 50 µL mixture of Matrigel and Intesticult™ medium in 24-well plates. 400 µL medium per well was used. On day 7 after isolation, the organoids were analyzed by light microscopy and qRT-PCR.

### ROS Measurements

HEK293 or CaCo2 cells were transiently transfected with plasmids (final concentration of 1.5 μg/3.5 cm dish) coding for the human sequence of Nox1, NoxO1, NoxA1, p47phox, p67phox, or GFP using lipofectamine 2000 (Thermo Fisher Scientific) according to the manufacturer’s instructions.

Reactive oxygen species production was assessed in intact cells and crypts. Measurement more specific for ${\text{O}}_{2}^{\cdot -}$ was carried out with L-012 (Wako Chemicals) (200 µmol/L) in a Berthold TriStar2 microplate reader (LB942, Berthold, Wildbad, Germany). All measurements were performed in HEPES-Tyrode buffer containing 137 mmol/L NaCl, 2.7 mmol/L KCl, 0.5 mmol/L MgCl_2_, 1.8 mmol/L CaCl_2_, 5 mmol/L glucose, 0.36 mmol/L NaH_2_PO_4_, 10 mmol/L HEPES. Activation of p47phox was triggered by phorbol myristate acetate (PMA, Sigma-Aldrich, 100 nmol/L). Superoxide dismutase (SOD, Sigma-Aldrich, 300 U/mL) was used to determine the specificity of the signal for ${\text{O}}_{2}^{\cdot -}$. H_2_O_2_ formation was measured with the Amplex Red assay (50 µM; Invitrogen, HRP, 2 U/mL, Sigma) as previously described (23). PEG-catalase (50 U/mL) served as a negative control.

For dihydroethidium (DHE)-based ROS detection, isolated crypts were incubated with DHE and DHE + 300 U/mL PEG-SOD for 30 min at 37°C in Hanks buffer containing 100 µmol/L diethylenetriaminepentaacetic acid pentasodium salt. For fluorescence detection, DHE and its oxidation products were separated by HPLC (Hitachi, Elite Lachrom system L3130 pump) using a C18 column (EC, Nucleosil, 100-5, 250/4.6 Macherey Nagel) and a mobile phase A of H_2_O:acetonitrile:TFA (9:1:0.1) and phase B of acetonitrile + 0.1% TFA. A gradient from 0 to 40% of B was achieved within 10 min and to 100% B in 20 min with a 0.5 mL/min flow. The oxidation products of DHE (40 µmol/L), 2-dihydroxyethidium (2EOH), and ethidium (E), were separated by HPLC and analyzed either by fluorescence 510 nm/595 nm excitation/emission for 2EOH in intact cells of crypts isolated from small intestine from different mouse strains.

### Analysis of mRNA Expression

Total mRNA from frozen homogenized tissue, isolated crypts, or cultured organoids were isolated with a RNA-Mini-kit (Bio&Sell, Feucht, Germany) according to the manufacturer’s protocol. Random hexamer primers (Promega, Madison, WI, USA) and Superscript III Reverse Transcriptase (Invitrogen, Darmstadt, Germany) were used for cDNA synthesis. Semi-quantitative real-time PCR was performed with AriaMx qPCR cycler (Agilent Technologie, Santa Clara, CA, USA) using iQ™ SYBR^®^ Green Supermix (BioRad, Hercules, CA, USA) with appropriate primers as listed below. Relative expression of target genes were normalized to eukaryotic translation elongation factor 2 or β-actin, analyzed by delta-delta-Ct method and represented as percentage of control samples.

**Table:** 

	Forward 3′–5′	Reverse 3′–5′
h,m,r EEF2	GACATCACCAAGGGTGTGCAG	GCGGTCAGCACACTGGCATA
m β-actin	TGACAGGATGCAGAAGGAGA	GCTGGAAGGTGGACAGTGAG
m Duox2	TCTTCACCATGATGCGGTCC	GGAGTCCGGTTGATGAACGA
m Lgr5	CCTACTCGAAGACTTACCCAGT	GCATTGGGGTGAATGATAGCA
m Nox1	CCTCCTGACTGTGCCAAAGG	ATTTGAACAACAGCACTCACCAA
m NoxA1	AGATACGGGACTGGCACCG	CATCCTAGCCAGCGGCTCTC
m NoxO1	ACTTAAACGCCTGTGCCATC	CCCCAACACTGCCCTAAGTA
m p22phox	TGTGGTGAAGCTTTTCGGGC	GGATGGCTGCCAGCAGATAGAT
m p47phox	TCCCAACTACGCAGGTGAAC	CCTGGGTTATCTCCTCCCCA
m p67phox	CTATCTGGGCAAGCCTACGGTT	CACAAAGCCAAACAATACGCG

### Protein and Western Blot Analysis

Samples were lysed using the following lysis buffer (pH 7.4, concentrations in mmol/L): Tris-HCl (50), NaCl (150), sodium pyrophosphate (10), sodium fluoride (20), nonidet P40 (1%), sodium deoxycholate (0.5%), proteinase inhibitor mix, phenylmethylsulfonyl fluoride (1), orthovanadate (2), and okadaic acid (0.00001). Then, they were cooked in Lämmli buffer and separated by SDS-PAGE followed by Western blotting. NoxO1 primary antibodies were made in our laboratory; LC3 antibody was obtained from MBL (#PM036); p62 antibody was obtained from Enzo (BML-PW9860-0100); and infrared-fluorescent-dye-conjugated secondary antibodies were obtained from Licor (Bad Homburg). Western blot analyses were performed with an infrared-based detection system (Odyssey, Licor, Bad Homburg, Germany).

### Statistics

Unless otherwise indicated, data are given as mean ± standard error of mean. Calculations were performed with Prism 5.0. Individual statistics of unpaired samples was performed by *t*-test and if not normal distributed by the Mann–Whitney test. A *p*-value of <0.05 was considered significant. Unless otherwise indicated, *n* indicates the number of individual experiments or animals.

## Results

### NoxO1 Is Highly Expressed in the Colon Epithelium

**Figure 1:**
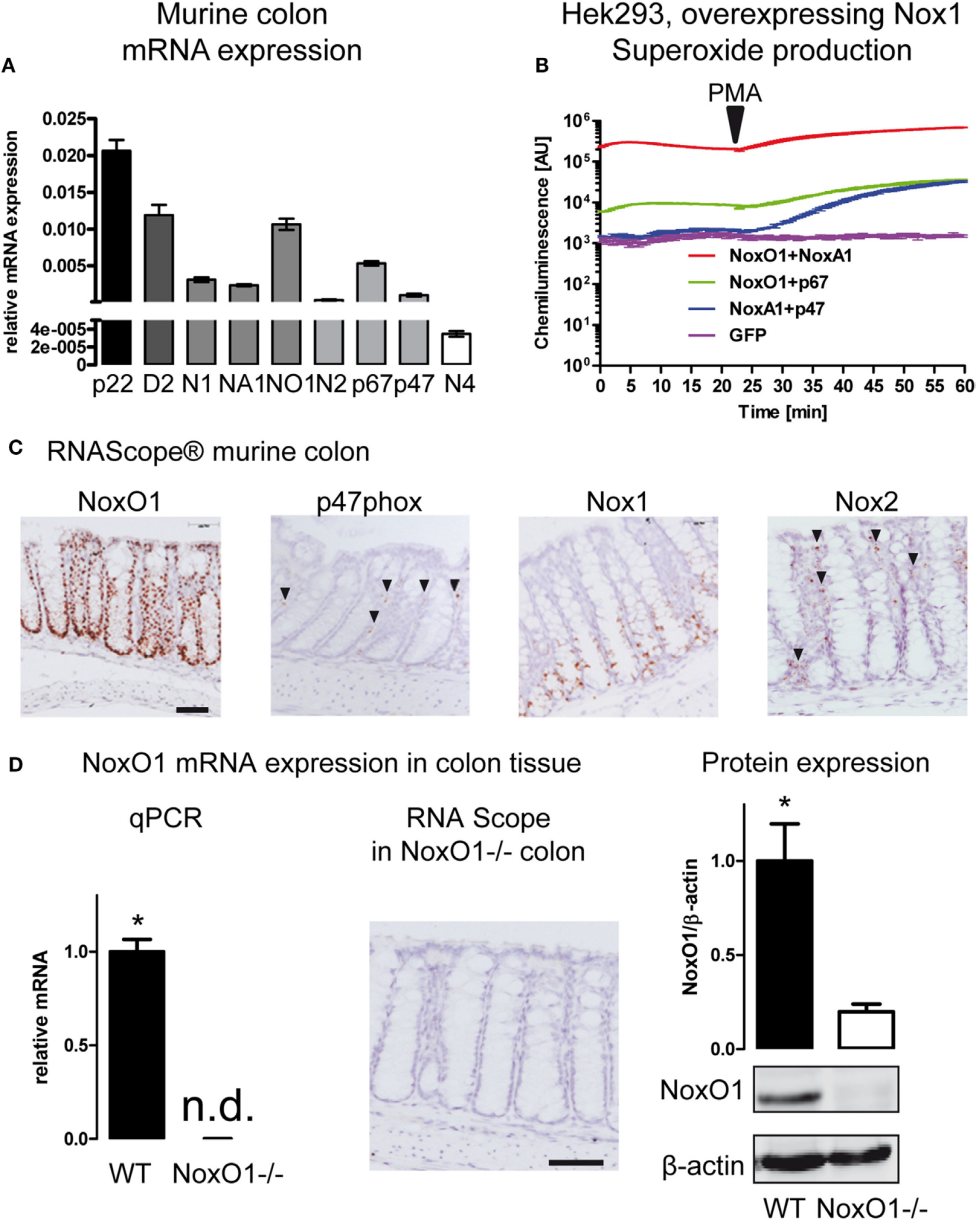
NADPH oxidase expression in the colon. **(A)** Analysis of mRNA expression in murine colon tissue. Relative expression to housekeeping gene EF. Genes indicated are p22phox (p22), Duox2 (D2), Nox1 (N1), NoxA1 (NA1), NoxO1 (N01), Nox2 (N2) p67phox (p67), p47phox (p47), and Nox4 (N4). *n* = 7–8. **(B)** HEK293 cells overexpressing Nox1 were transfected with cytosolic subunits of the NADPH oxidase complex as indicated. Reactive oxygen species were measured with L012 (200 μmol/L). Activation of p47phox was triggered by phorbol myristate acetate (100 nmol/L). **(C)** *In situ* hybridization (RNAScope^®^ DAB staining) showing the expression of NADPH oxidase subunits NoxO1, p47phox, Nox1, and Nox2 in murine colon tissue. Nuclei were counterstained with hematoxylin. Scale bars indicate 100 μm. **(D)** Quantitative RT-PCR, RNAScpoe^®^, and immunoblotting analysis of Nox1 protein expression and in colon tissue of wild-type (WT) and NoxO1 knockout (NoxO1^−/−^), *n* = 6, *p < 0.05 WT vs. NoxO1^−/−^.

The expression of members of the NADPH oxidase family in the colon was analyzed by qRT-PCR (Figure 1A; Figures S1 and S2 in Supplementary Material). The scaffolding component of the NADPH oxidase complex p22phox showed the highest expression level, followed by Duox2 and NoxO1, Nox1, and the activating subunit NoxA1. Furthermore, the activating subunits of Nox2, p47phox, and p67phox, which serve as organizer and activator of the Nox2 enzyme complex are expressed at relatively high levels in the colon. In an overexpression system, p47phox and p67phox can substitute NoxO1 and NoxA1 in the Nox1 complex and *vice versa*. In such a system, Nox1 together with NoxA1 and NoxO1 produced a large amount of ${\text{O}}_{2}^{\cdot -}$ in a constitutive manner, while the complex of Nox1 with NoxO1/p67phox produces constitutively lower levels of ${\text{O}}_{2}^{\cdot -}$. The combination of p47phox/NoxA1 under basal conditions does not change the level of ${\text{O}}_{2}^{\cdot -}$. Upon stimulation with PMA, p47phox is activated, and ROS formation increases to the level of the Nox1/p67phox/NoxO1 complex (Figure 1B). Overexpression of Nox1 or NoxO1 alone in CaCo cells revealed much lower ${\text{O}}_{2}^{\cdot -}$ formation than overexpression of all three components of the complex (Figure S2C in Supplementary Material). Importantly, when measuring mainly H_2_O_2_ with the aid of Amplex Red, overexpression of neither the single nor the combination of the plasmids revealed any difference in the formation of H_2_O_2_ (Figure S2D in Supplementary Material). Those results indicate that indeed, NoxO1, together with Nox1 and NoxA1, contributes to a constant ${\text{O}}_{2}^{\cdot -}$ formation in colon crypts. To explore a potential substitution of NoxO1 by p47phox in the colon, we analyzed the localization of the NADPH oxidase components in the colon (Figure 1C). For that purpose, *in situ* hybridization by RNAScope^®^ was performed in colon Swiss rolls from WT animals. As shown for the vascular system (24), also in the colon p47phox does not occur in the same cells as NoxO1 and therefore may have totally distinct roles than NoxO1 in the colon. Rather than in epithelial cells, p47phox was located in capillaries, while the expression of NoxO1 was strongly restricted to epithelial cells. We confirmed that Nox1 mRNA expression is present in the lower two thirds of the colon crypts (25). Expression of NoxO1 in WT and NoxO1^−/−^ colons was analyzed by qRT-PCR and RNAScope, while protein expression of NoxO1 was analyzed by Western blot (Figure 1D).

### Knockout of NoxO1 Leads to Loss of Superoxide Production

**Figure 2:**
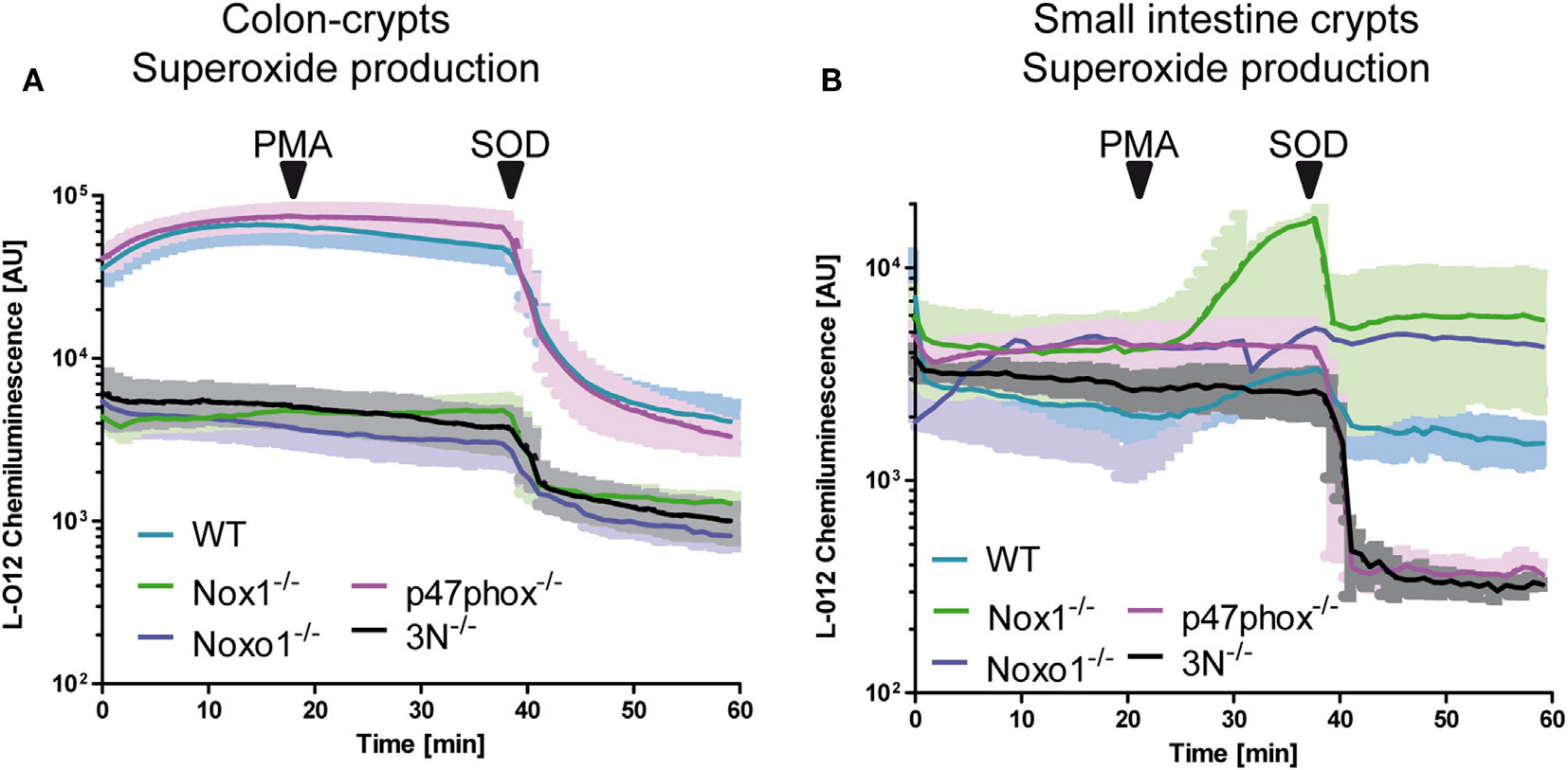
NoxO1-dependent reactive oxygen species (ROS) formation in colon crypts. A + B Crypts from colon **(A)** or small intestine **(B)** were isolated. ROS were measured by chemiluminescence with L-012 (200 μmol/L). Activation of p47phox was triggered by phorbol myristate acetate (PMA, 100 nmol/L). Superoxide anions were decomposed with superoxide dismutase (SOD, 300 U/mL); *n* = 4.

Whether or not Nox1 and NoxO1 form a functional complex in colon crypts was analyzed *via* the measurement of ROS in isolated colon crypts (Figure 2A). To simultaneously analyze, if there is a functional substitution for the lack of NoxO1 by p47phox, we also used isolated crypts from p47phox^−/−^ mice. ROS were analyzed using L-012 chemiluminescence and DHE, to concentrate more on the formation of ${\text{O}}_{2}^{\cdot -}$ than of H_2_O_2_, and crypts from mice with a knock out for Nox1, Nox2, and Nox4 (3N^−/−^) were analyzed to learn what would be the basal level of NADPH oxidase-independent production of ${\text{O}}_{2}^{\cdot -}$. Knockout of Nox1 and NoxO1 drastically reduced the formation of ${\text{O}}_{2}^{\cdot -}$, while the deletion of p47phox had no effect on the formation of ROS in colon crypts. Importantly, triple knockout of Nox1, Nox2, and Nox4 reduced the ${\text{O}}_{2}^{\cdot -}$ formation to the level of Nox1 or NoxO1 knockout. Stimulation of the ROS formation with the p47phox activator PMA had no effect on ROS formation in colon crypts, but in crypts from the duodenum (Figure 2B). ROS formation in the duodenum crypts is 10× lower than in colon. Obviously, the basal duodenal ROS formation is Nox independent but SOD sensitive, as analyzed by a DHE-based measurement of ${\text{O}}_{2}^{\cdot -}$ (Figure S2B in Supplementary Material). This is a reflection of the fact that NoxO1 expression is high in the colon and masks the low PMA-activated ROS formation by p47phox. In fact, the difference in ROS formation corresponds to the expression of NoxO1 (1 ± 0.1 in the duodenum vs. 9.1 ± 0.6 in the colon).

### NoxO1 Affects Proliferation and Differentiation *In Vivo*

**Figure 3:**
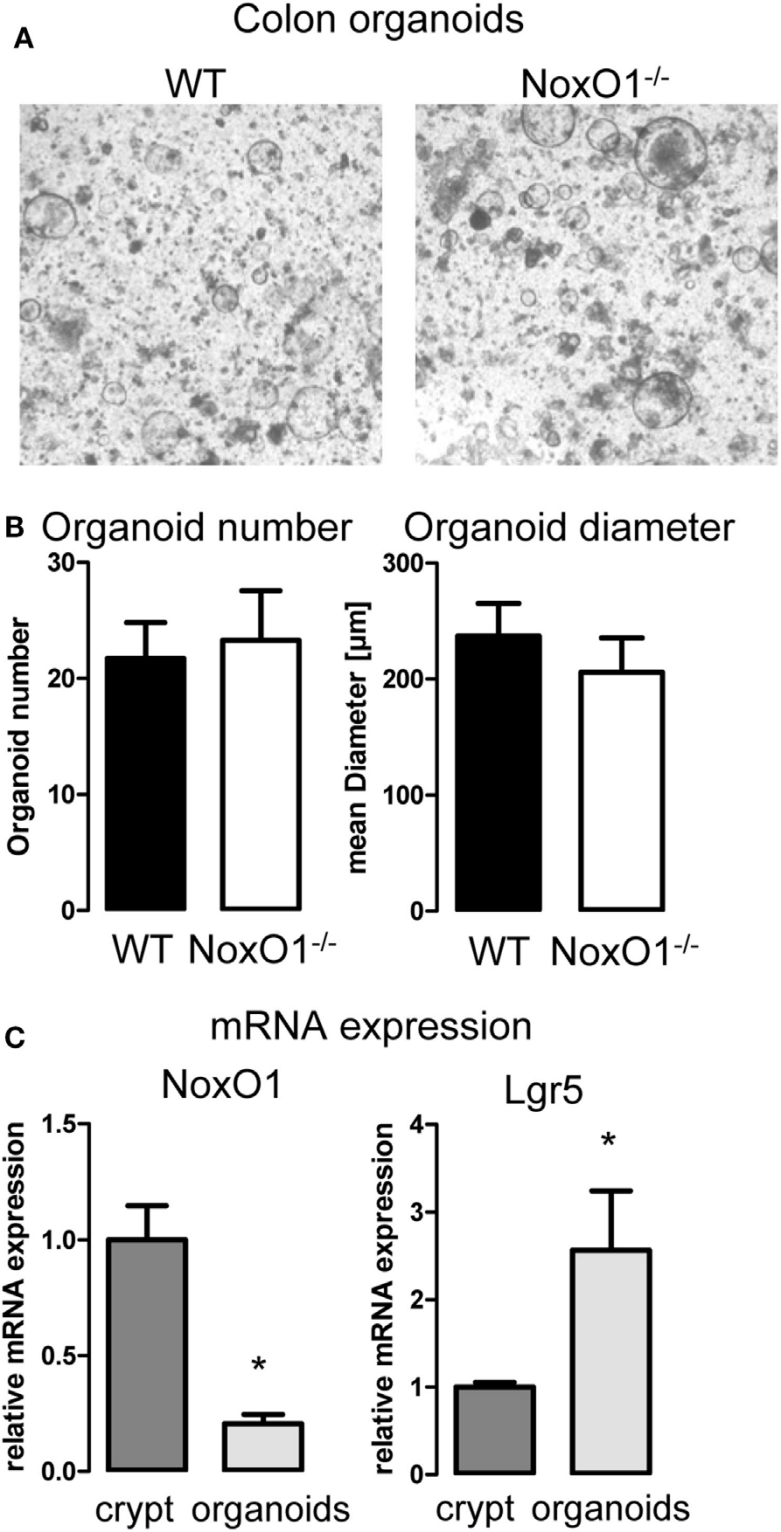
Colon organoid growth. **(A)** 3D culture of organoids generated from colon crypts isolated from wild-type (WT) and NoxO1 knockout (NoxO1^−/−^) animals. Representative pictures of organoids after 7 days in culture. **(B)** Analysis of organoid number and mean diameter of organoids cultured for 7 days. Organoids were counted per field of vision. Per animal two wells were analyzed. *n* = 5. **(C)** mRNA expression relative to housekeeping gene EF of NoxO1 and stem cell marker Lgr5 in freshly isolated crypts and organoids cultured for 7 days. *n* = 3, *p < 0.05.

As pointed out already, ROS affect proliferation. So far, no protocol has been successfully established for a primary culture of colon epithelial cells. Therefore, to analyze the role of NoxO1 in proliferation and differentiation independent from pathogens or gut flora and food intake, 3D organoid cultures from colon crypts were established (Figure 3A). The number of organoids indicates the potential of the crypts to form organoids, while organoid diameters correspond to the proliferative capacity of the cells. Unexpectedly, no difference in both parameters was observed when comparing WT and NoxO1^−/−^ organoids (Figure 3B). These puzzling data were clarified when comparing freshly isolated colon crypts and established colon organoids, which revealed a reduction of NoxO1 expression by 80%. In contrast, the stem cell marker Lgr5 was increased more than twofold (Figure 3C). This indicates, although stemness is maintained in the organoids, that NoxO1 expression is drastically reduced and therefore does not impact proliferation.

**Figure 4:**
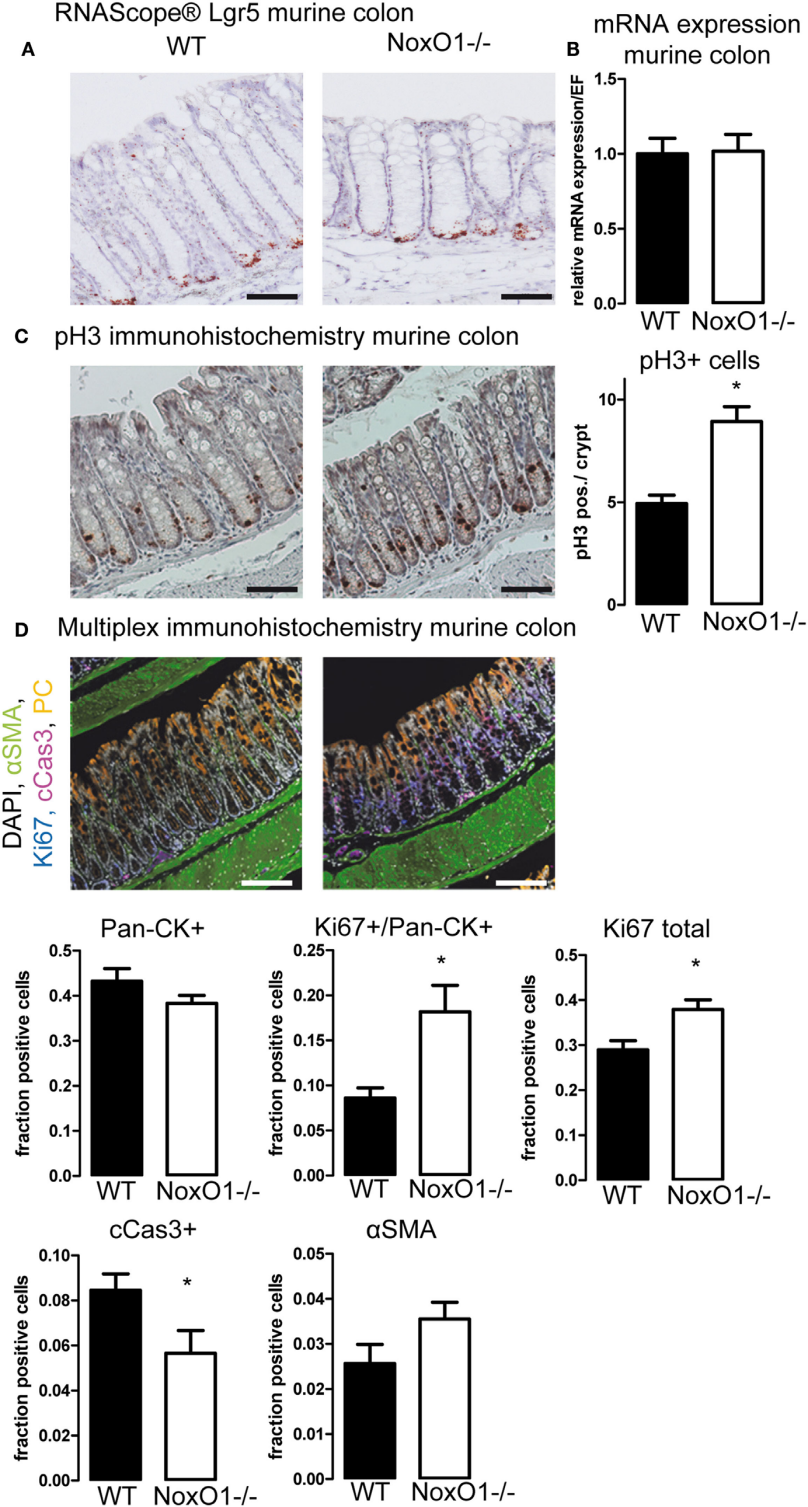
Immunohistochemistry of colons. **(A)** *In situ* hybridization (RNAScope^®^) of Lgr5 in murine colon. Nuclei were counterstained with hematoxylin, scale bars indicate 100 μm **(B)** Lgr5 mRNA expression relative to housekeeping gene EF in colon tissue from wild-type (WT) and NoxO1 knockout (NoxO1^−/−^) mice. *n* = 7. **(C)** Immunohistochemistry staining of mitosis marker phospho-Histone 3 (pH3). Representative pictures and analysis of pH3-positive cells per crypt. *n* = 3, *p < 0.05. Scale bars indicate 200 μm. **(D)** Multiplex immunohistochemistry of murine colon. Tissue was stained for DAPI (white), alpha-smooth muscle actin (aSMA, green), Ki67 (blue), cleaved Caspase 3 (cCas3, magenta), and pan-cytokeratin (Pan-CK, orange); scale bars indicate 100 μm. Positive cell fractions for indicated phenotypes in the mucosa were analyzed using the in Form 2.0 software. *n* = 5, *p < 0.05.

Having found that analysis of the influence of NoxO1 on proliferation *in vitro* was not possible, we went on to the analyses of *in vivo* samples by immunohistochemistry. Staining for the stem cell marker Lgr5 by *in situ* hybridization (Figure 4A) and analysis of its mRNA expression (Figure 4B) indicated no difference between the stem cell potential in crypts from WT and NoxO1 knockout mice. Staining for pH3, an indicator of active mitosis, indicated a higher proliferative activity in the absence of NoxO1 (Figure 4C). Importantly, no effect of NoxO1 knock out was found when we analyzed for autophagy (Figure S3 in Supplementary Material). Subsequently, the cells in the mucosa were phenotyped according to their marker expression (Figure 4D). Alpha-SMA-positive cells represent smooth muscle cells and were found below and between the crypts. Pan-cytokeratin is a marker for epithelial cells and was used for the definition of differentiated cells. The expected gradient of pan-cytokeratin was observed along the crypt axis, with an increase at the top of the crypt. While not significant, a trend indicated a reduced differentiation of the crypt cells in the absence of NoxO1. Ki67 is an established marker for proliferating cells. Although about 50% of all cells in the mucosa were pan-cytokeratin (Pan-CK) positive, these cells still may proliferate and therefore are Ki67/Pan-CK double positive. Both pan-CK/Ki67 double positive cells and total Ki67 staining were increased in colons from mice deficient in NoxO1 (Figures 4C,D). Interestingly, cells undergoing apoptosis and positive for cleaved caspase 3 were reduced in NoxO1-deficient colons. Together, the results suggest that the absence of NoxO1 results in a diminished differentiation, more proliferation, and less apoptosis in epithelial cells of colon crypts.

### NoxO1 Has a Protective Role in DSS-Induced Colitis

The combination of less differentiation, less apoptosis, and more proliferation results in more S-phase cells, which are sensitive to DNA damage. This may increase the risk of neoplastic transformation and enhanced tumor incidence (2).

**Figure 5:**
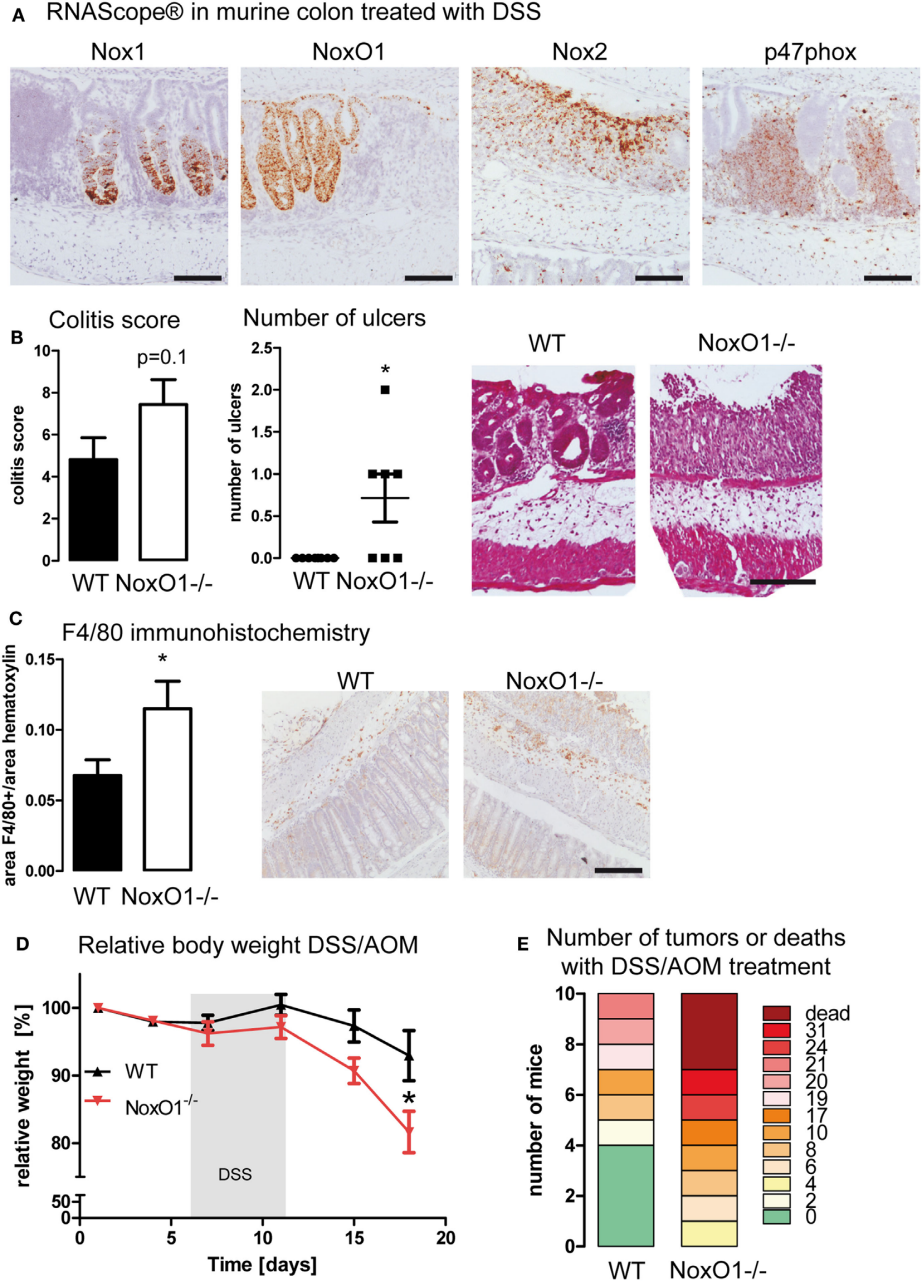
NoxO1 has a protective role in dextran sulfate sodium (DSS)-induced colitis and DSS/azoxymethane (AOM) colon carcinoma. **(A)** *In situ* hybridization (RNAScope^®^) showing the expression of Nox1, NoxO1, Nox2, and p47phox in murine colon tissue upon treatment with DSS. Nuclei were counterstained with hematoxylin, scale bars indicate 100 μm. **(B)** Quantification of histological damage and number of ulcers on day 8 of the colitis model. *n* = 7–8, *p < 0,05, Mann–Whitney exact test. Representative pictures of hematoxylin and eosin (H&E) staining for wild-type (WT) and NoxO1^−/−^ are shown. Scale bar indicates 200 μm. **(C)** Immunohistochemistry staining of macrophage marker F4/80. Representative pictures and analysis of F4/80-positive staining per nuclei. *n* = 7–8, *p < 0.05. Scale bars indicate 500 μm. **(D)** Body weight relative to day 0 of WT and NoxO1^−/−^ mice treated with AOM at day 0 and 3 cycles of DSS in drinking water. *n* = 9, *p < 0.05. **(E)** Tumor burden at day 70 of DSS/AOM-treated animals. *n* = 8–10.

To analyze this possibility, mice were subjected to the DSS-induced colitis and DSS/AOM colon cancer model. RNAScope^®^ analysis in colon samples upon DSS treatment verified that Nox1 and NoxO1 expression remains only in the epithelium, whereas Nox2 and p47phox were expressed in infiltrating immune cells (Figure 5A). To analyze disease severity, the inflammatory damage score was analyzed. Crypt damage, immune cell infiltration, and development of ulcers were more severe in NoxO1^−/−^ mice (Figure 5B). This was also confirmed by immunohistochemical staining of macrophages by F4/80 (Figure 5C). Analysis of immune cell populations did further unmask a difference in the content of natural killer cells in DSS-treated mice (NK cells as percentage of all immune cells: 5.6 ± 1.2 WT ctl; 6.6 ± 0.6 NoxO1^−/−^ ctl; 8.0 ± 0.7 WT DSS; 4.8 ± 0.8* NoxO1^−/−^ DSS; *p < 0.05 WT DSS vs. NoxO1^−/−^ DSS). In contrast, in the tumor AOM/DSS model, less macrophages were found in NoxO1^−/−^-deficient tumors, as analyzed by conventional immunohistochemistry (Figure S4A in Supplementary Material) and a costaining of F4/80 (Adgre1) and the Noxes on mRNA level (Figure S4B in Supplementary Material). Interestingly, macrophage infiltration into colon tumors after an initial increase appears to decrease after a while (26). This effect of a reduced content of F4/80-positive cells at day 70 can be seen in NoxO1^−/−^ mice as well (Figure S4 in Supplementary Material). DSS/AOM treatment induced a loss of body weight, indicating the severity of the inflammation due to DSS. Weight loss was more severe in NoxO1^−/−^ mice (Figure 5D). Eventually, although not significant, there was a trend for a higher tumor burden and mortality, in NoxO1-deficient animals when compared to their WT littermates (Figure 5E; Figures S4C,D in Supplementary Material).

## Discussion

The colon epithelium is constantly renewed and arises from only a few intestinal stem cells residing at the crypt base. From these amplifying cells, the epithelial cell layer derives that progressively differentiates until the top of the villi. This process is redox sensitive. Especially, Nox1-derived ROS may influence the balance of proliferation and differentiation in the gut epithelium. Nox1 upregulation enhances Wnt/β-catenin and Notch pathways and disrupts tumor progression by pro-apoptotic mechanisms. An excellent review on redox signaling in the gastrointestinal tract has been published by Pérez et al. (27).

While Nox1 indeed is the central molecule of the NADPH oxidase in the colon, the cytosolic subunits determine the signaling mediated by Nox1-derived ROS. Those cytosolic subunits are the activators p67phox or NoxA1 and the organizers p47phox and NoxO1. In an overexpressing system, the combination of NoxO1/p67phox and NoxO1/NoxA1 together with Nox1 enables a constitutive formation of superoxide, while the combination of Nox1 with NoxA1 and p47phox enables an acutely inducible ROS formation. This is due to the fact that NoxO1 is missing the autoinhibitory loop, which prevents the activity of p47phox. Upon serine phosphorylation, p47phox is activated and organizes the Nox1 complex. Therefore, p47phox-mediated ROS formation can be switched on and off (16). Consequently, p47phox may contribute to acute ROS-sensitive signaling, while NoxO1 serves as a mediator of constant redox-dependent signaling, such as differentiation, proliferation, or survival of a cell. In fact, NoxO1 and p47phox appear to have totally distinct functions and do not substitute for each other *in vivo* (24, 28). The current study indicates that the distinct expression site and the behavior of not subsidizing for each other are also true for the intestinal system. However, the role of NoxO1 in the gut was uncertain. In endothelial cells, NoxO1 is needed to maintain the activity of the Notch signaling pathway by enabling the activity of a disintegrin and metalloproteinases (ADAM) as measured in an ADAM10/17 activity assay. In contrast, this task was not fulfilled by p47phox (17). Notch also plays an extraordinary important role in the renewal of the colon epithelium, and ADAM10 is abundantly expressed throughout the gastrointestinal tract. During intestinal renewal homeostasis, ADAM10 regulates cellular processes such as cell fate specification and maintenance of intestinal stem cell/progenitor populations, controlling intestinal injury/regenerative responses and may drive intestinal inflammation and colon cancer initiation and progression (29). Therefore, a role of NoxO1 in all these processes is possible. In fact, proliferation was enhanced, while apoptosis was reduced in the absence of NoxO1 in murine colons. Those data perfectly fit an earlier study showing that Nox1 is required for reconstitution of the epithelium after colitis induction (10), indicating that NoxO1 together with Nox1 mediates ROS formation and facilitates proliferation of colon epithelial cells. Colitis as such, however, appears to be more severe in the absence of NoxO1. This could be a consequence of a reduced number of natural killer cells. Importantly, the activity of natural killer cells has been found to be significantly below normal levels in both remissive and active stages of inflammatory bowel disease patients (30). In fact, natural killer cells protect mice from DSS-induced colitis by regulating neutrophil function *via* the NKG2A receptor (31). Why less natural killer cells are present in DSS-treated NoxO1^−/−^ mice is beyond the scope of this manuscript. One possibility is that NoxO1 is essential for the differentiation of those cells from their progenitors (24). Nevertheless, inflammatory bowel disease patients have a higher incidence of cancer (32). A similar effect was found in this study. The absence of NoxO1 increased the likelihood for tumor development and the number of tumors in the AOM/DSS colon cancer model. This is potentially a consequence of the increased DSS-induced inflammation in the absence of NoxO1, together with an enormous proliferation and reduction in apoptosis of epithelial cells. This combination is prone to support malignant transformation and the development of tumors (33).

In conclusion, NoxO1 contributes to a constitutive ROS formation in colon epithelial cells. NoxO1 cannot be substituted by p47phox. NoxO1 limits proliferation and increases the ability of epithelial cells to undergo apoptosis. Colitis, as induced by DSS, is more severe in the absence of NoxO1 in mice. Eventually, while deserving further studies, in an AOM/DSS murine colon cancer model NoxO1 appeared to be protective.

## Ethics Statement

All animal experiments were approved by the local governmental authorities (approval number: FU1074, F28/46) and were performed in accordance with the animal protection guidelines.

## Author Contributions

All the authors performed experiments, collected or analyzed data, and helped with techniques. FM and KS wrote the manuscript.

## Conflict of Interest Statement

The authors declare that the research was conducted in the absence of any commercial or financial relationships that could be construed as a potential conflict of interest. The reviewer GC and handling Editor declared their shared affiliation.
