# Supplementary figures and images for: NoxO1 Controls Proliferation of Colon Epithelial Cells
Source: Front Immunol. 2018 May 8;9:973. doi: 10.3389/fimmu.2018.00973 (PMC5951971; doi:10.3389/fimmu.2018.00973)

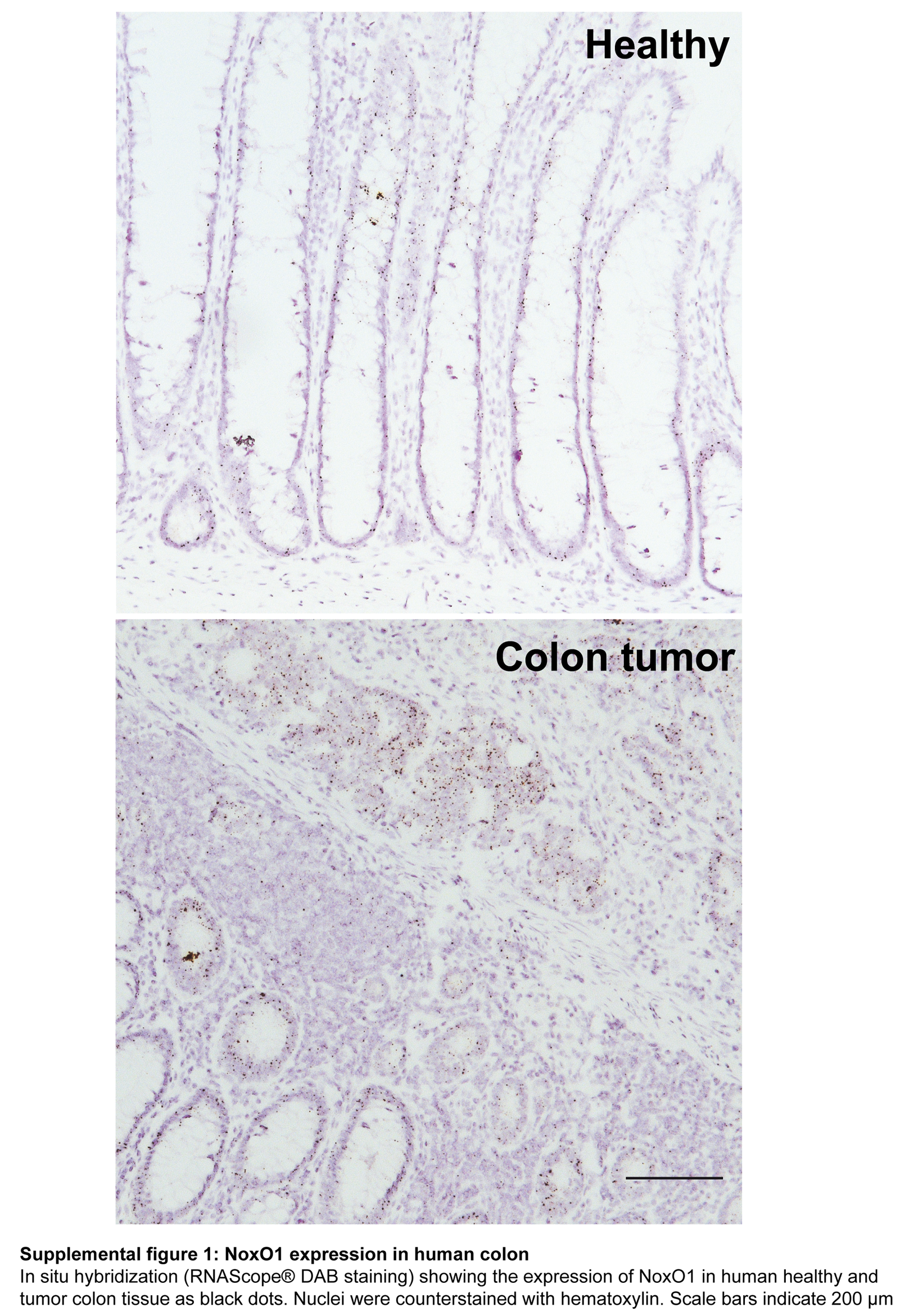

Supplement: Supplementary file 1 [file image_1.tif]

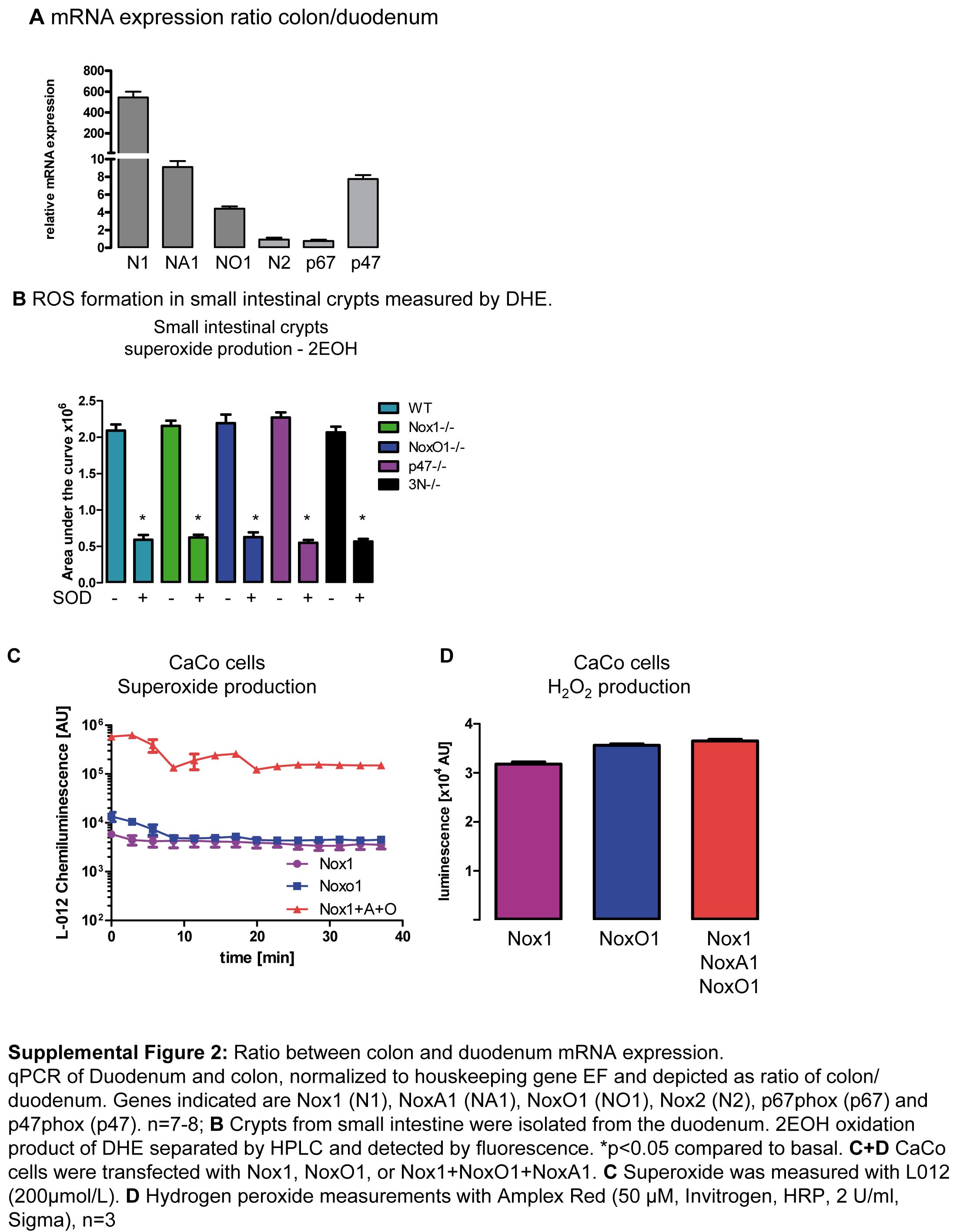

Supplement: Supplementary file 2 [file image_2.tif]

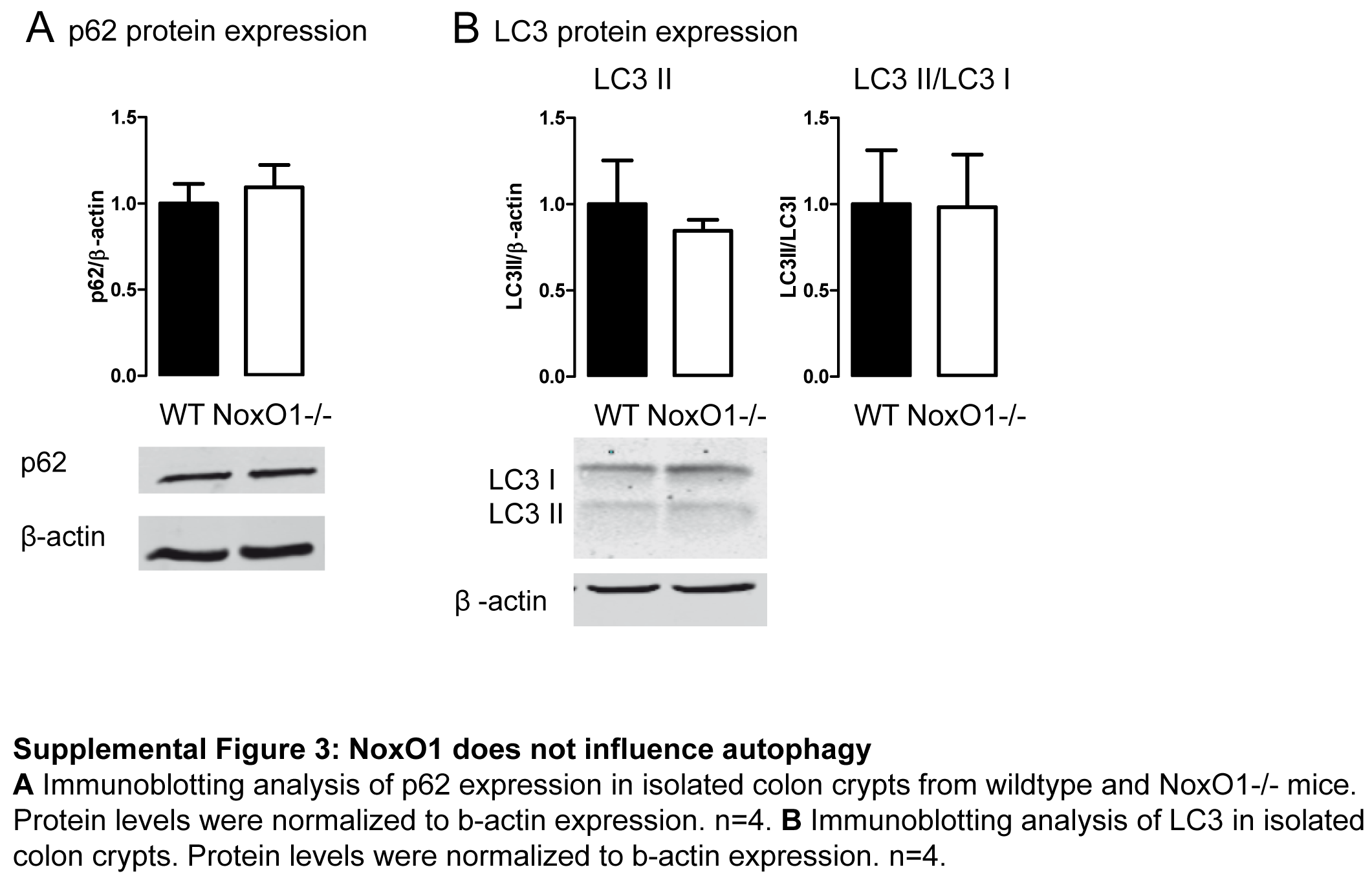

Supplement: Supplementary file 3 [file image_3.tif]

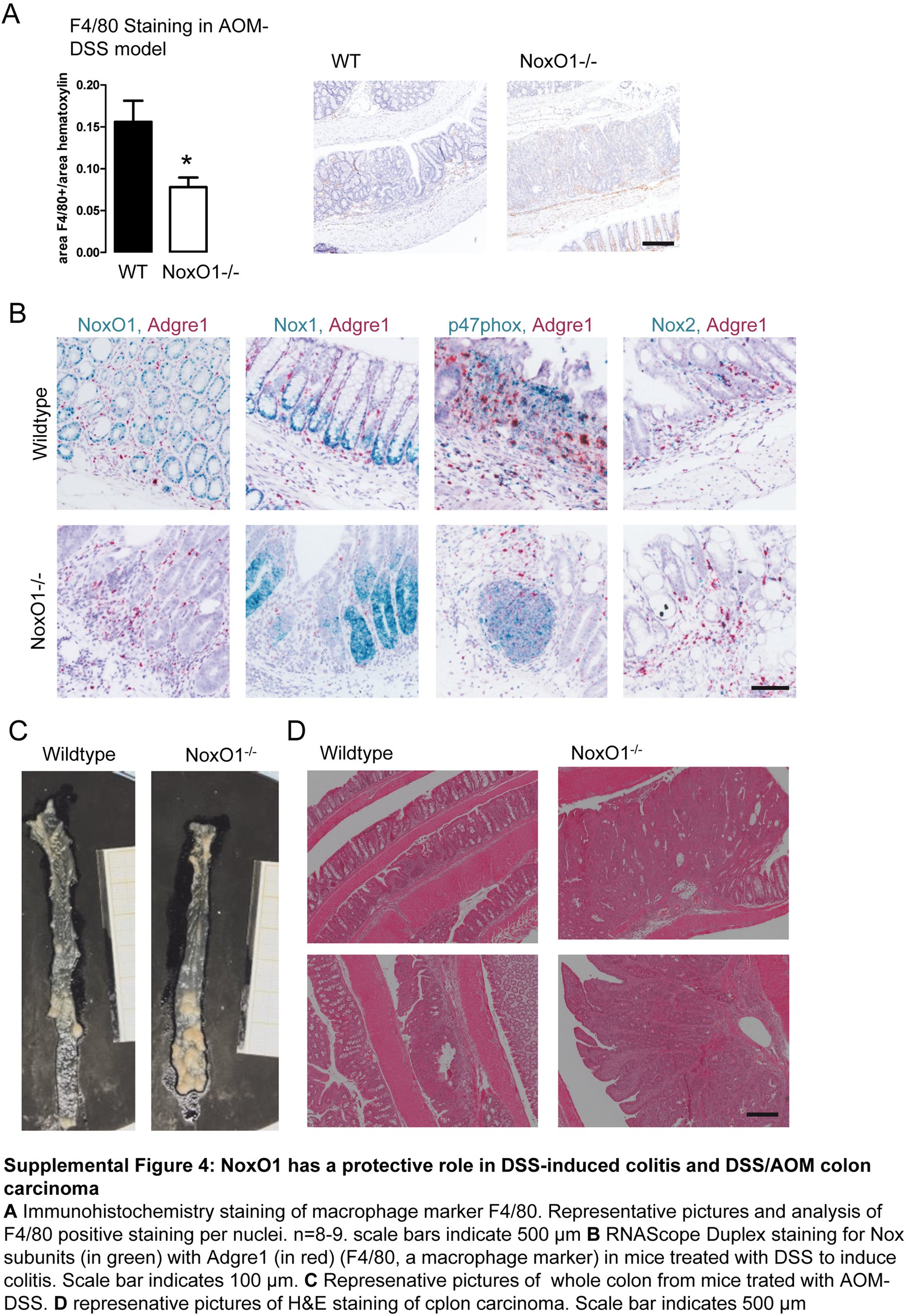

Supplement: Supplementary file 4 [file image_4.tif]
